# The prognostic value of p62 in solid tumor patients: a meta-analysis

**DOI:** 10.18632/oncotarget.23101

**Published:** 2017-12-07

**Authors:** Haihua Ruan, Jingyue Xu, Lingling Wang, Zhenyu Zhao, Lingqin Kong, Bei Lan, Xichuan Li

**Affiliations:** ^1^ Tianjin Key Laboratory of Food Science and Biotechnology, College of Biotechnology and Food Science, Tianjin University of Commerce, Tianjin, China; ^2^ Department of Clinical Laboratory, The Fifth Central Hospital of Tianjin, Tianjin, China; ^3^ School of Basic Medical Sciences, Tianjin Medical University, Tianjin, China; ^4^ Department of Pharmacy, Tianjin Medical University Metabolic Disease Hospital, Tianjin, China; ^5^ Jining Tumor Hospital, Jining No.1 People's Hospital North Campus, Shandong, China

**Keywords:** solid tumors, p62, prognosis, meta-analysis

## Abstract

p62, as a scaffolding/adaptor protein, is involved in multiple physiological processes include inflammation, autophagy and mitosis. However, the influence of p62 in cancer patients has not been comprehensively investigated. Moreover, the prognostic value of p62 for the survival of patients with solid tumors remains controversial. In this present meta-analysis, twenty suitable articles were identified from PubMed, EMBASE and Web of Science, Nature databases, including 4271 patients. A random-effect or fixed-effect model was adopted to correlate p62 expression with different outcome measured in entire tumors. Combined with results of hazard ratios (HRs) and 95% confidence intervals (CIs), we concluded that higher expression of p62 is associated with poorer overall survival (OS) (HR: 2.22, 95% CI: 1.82–2.71, *P* < 0.05), disease-free survival (DFS) (HR = 2.48, 95% CI: 1.78–3.46, *P* < 0.05) and even certain clinicopathological parameters, such as lymph node metastasis (RR = 1.21, 95% CI: 1.06–1.37) and clinical stages (RR = 1.27, 95% CI: 1.12–1.45), in cancer patients. Consequently, our data showed that p62 might be an effective poor prognostic factor for patients with various solid tumors.

## INTRODUCTION

p62 (sequestosome-1) was first identified as an interaction protein with human p56lck SH domain [1]. Distinct from human, murine p62 homolog ZIP was independently identified as a binding partner of atypical protein kinase C (PKC-ζ) [2]. A plenty of studies have found that p62 is a scaffold protein and is involved in several important signal pathways like NF-κB signaling, autophagy, mitosis to influence inflammation, deoxidization, cell growth, and cell cycle, which may affect tumorigenesis [3, 4].

p62 serves as a crucial factor during the process of tumorigenesis. Firstly, p62 down-regulates the level of reactive oxygen species (ROS) to promote tumorigenesis. p62 consists of multiple domains: the Phox1 and Bem1p (PB1) domain, that can interact with PKC-ζ, the ZZ-type zinc finger (ZZ) domain, that interact with receptor-interacting protein (RIP), the TB domain, that interact with tumor necrosis factor receptor-associated factor 6 (TRAF6), the light chain 3-interacting region (LIR) domain that interact with light chain 3 (LC3) and the ubiquitin-associated (UBA) domain that binding ubiquitin. The interactions between p62 with PKC-ζ, RIP and TRAF6 can activate NF-κB signaling [5, 6], which leads to down-regulation of ROS, thus avoiding the initiation of apoptosis pathway and promoting carcinogenesis [7]. Additionally, Keap1-Nrf2 complex is a key component to response to cellular oxidative stress [8], p62 directly interacts with Keap1 to prevent Nrf2 from being degraded by ubiquitin-proteasome system, subsequently allow Nrf2 to activate the downstream antioxidant genes to suppress ROS level [9–11]. Secondly, p62 exhibit non-liner, complicate interaction with autophagy to influence carcinogenesis process [3]. p62 can recruit Raptor and Rags proteins to activate mTORC1 and consequently inhibit autophagy [12]. Additionally, p62 is also a substrate of autophagy [13], thus the up-regulation of autophagy will decrease p62 level, which further activates autophagy. This feed forward loop may ensure the irreversible activation of autophagy under nutrient deprivation. Researchers have also found impaired autophagy can induce the accumulation of p62 to promote carcinogenesis [14]. In mitosis, constitutive phosphorylation of p62 T269 and S272 residues by Cdk1 can prevent cell from carcinogenesis. Mutant p62 that cannot be phosphorylated on those two residues will faster exit mitosis and increase cell proliferation, which promotes tumorigenesis in Ras-transformed cells [15].

Although much relationship between p62 and tumorigenesis has been unveiled in laboratory, it is unclear the actual prognosis value of this adaptor protein in cancer patients. Does p62, a hub of NF-κB, autophagy and mitosis pathway, can serve as a stable cancer biomarker? The aim of our meta-analysis is to give a quantitative assessment to the prognostic value of p62 in various type of cancer.

## RESULTS

### Study selection and characteristics description

**Figure 1:**
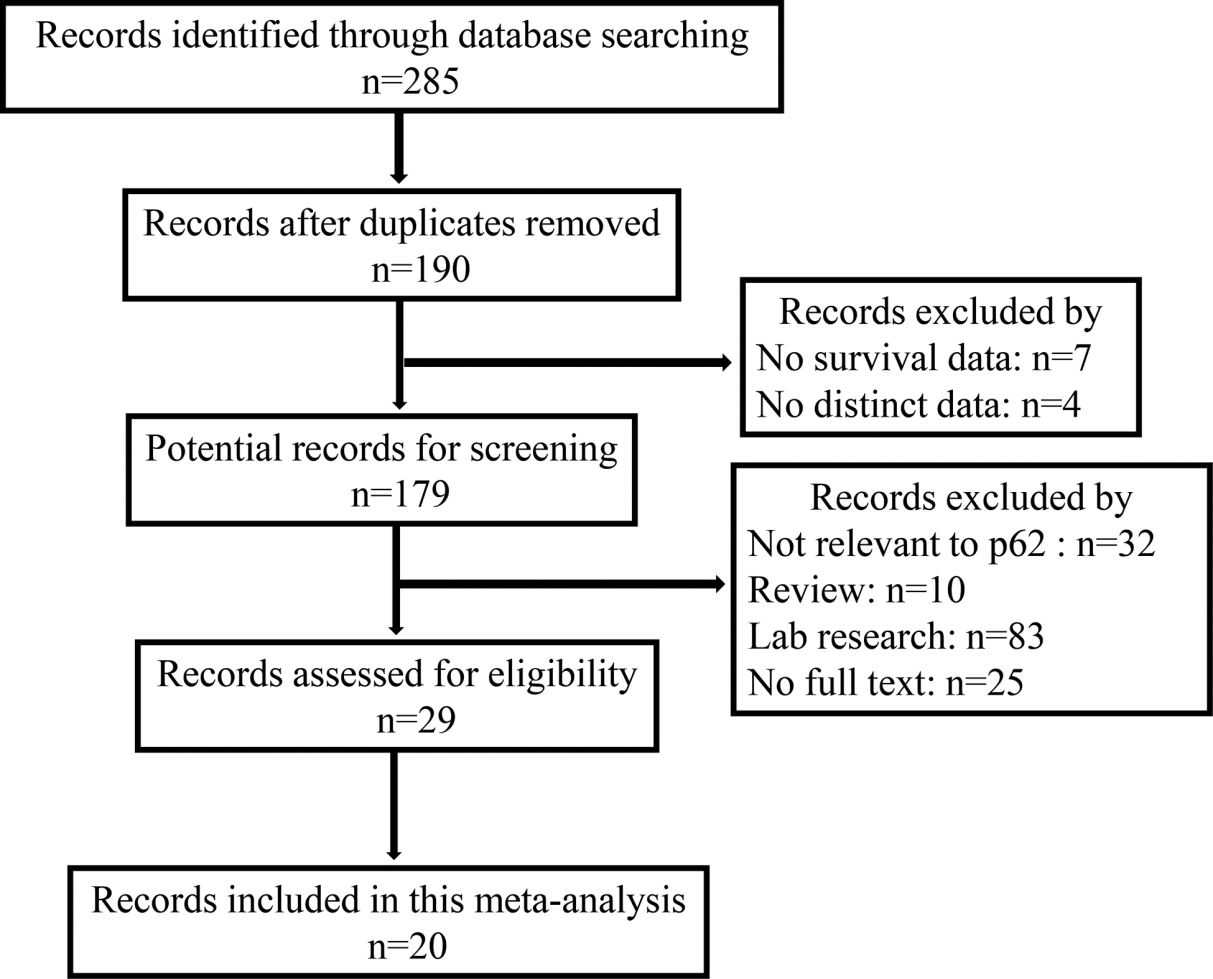
Flow diagram of the selection of eligible studies

The databases including PubMed, EMBASE and ISI Web of Science, were originally searched for a total of 285 articles containing keywords p62 and prognosis. 265 of those were excluded, due to repetitive researches (*n* = 95), without full texts (*n* = 25), laboratory studies (*n* = 103), reviews (*n* = 10), studies not relevant to the current analysis (*n* = 32). 20 publications [16–35] were selected for this meta-analysis containing 4271 patients. The flow chart of the study search and selection process is reported in Figure 1. The articles to be selected were collected as of September 2017. Immunohistochemistry (IHC) was the only method to evaluate p62 expression in human specimens. The main tumor types of these patients are breast cancer (*n* = 1474), non-small lung cancer (*n* = 659) and melanoma (*n* = 196). In addition, > 10% positive tumor cells and scores greater than one were the most suitable cut-off values for overall survival, > 10% positive tumor cells were a more suitable cut-off value for disease-free survival at the same time. The main characteristics of the 20 included studies are summarized in Table 1. This meta-analysis was performed with the guideline of Cochrane.

**Table 1:** Main characteristics of studies exploring the relationship between p62 expression and tumor prognosis

Author	Year	Region	Cancer Type	No. of Patients	Follow-up Time Median (range)	Detection Method	Cut-off	Outcomes	NOS Score
Shun Nakayama [16]	2017	Japan	Colorectal Carcinoma	118	69.8 m (2–131)	IHC (MBL)	≥ 10%	OS	7
Akihito Arai [17]	2017	Japan	Hypopharyngeal Carcinoma	54	NR	IHC (MBL)	NR	DFS	5
Diana Y. L. Tang [18]	2016	UK	Melanoma	75	5 y	IHC (NR)	≥ 20%	OS, DFS	6
Xifeng Wang [19]	2015	China	Non-Small Cell Lung Cancer	104	48.5 m (3–96.5)	IHC (Abcam)	IRS ≥ 4	OS	7
Reiko Iwadate [20]	2015	Japan	Endometrial Cancer	194	22.0 m (2.0–58.0)	IHC (Santa Cruz)	≥ 10%	OS	8
Mingfei Zhao [21]	2015	China	Gliomas	75	24 m (6–60)	IHC (Santa Cruz)	IRS ≥ 3	OS, DFS	6
Xianhan Jiang [22]	2015	China	Prostate Cancer	149	10 y	IHC (Santa Cruz)	IRS ≥ 4	OS	7
J-L Liu [23]	2014	China	Oral Squamous Cell Carcinoma	195	47.08 ± 32.37 m	IHC (Abcam)	IRS ≥ 4	OS, DFS	8
Reiko Iwadate [24]	2014	Japan	Epithelial Ovarian Cancer	266	59 m (1–120)	IHC (Santa Cruz)	≥ 10%	OS	8
Robert A Ellis [25]	2014	UK	Melanoma	121	7 y	IHC (NR)	≥ 20%	DFS	7
Sang Kyum Kim [26]	2013	Korea	Phyllodes Tumor	190	NR	IHC (Abcam)	IRS ≥ 2	OS, DFS	7
Rong-Zhen Luo [27]	2013	China	Breast Cancer	163	112 m (15–145)	IHC (Santa Cruz)	IRS ≥ 2	OS, DFS	7
Jae Myung Park [28]	2012	USA	Colon Carcinoma	178	4 y	IHC (MBL)	≥ 50%	OS	7
Junjeong Choi [29]	2012	Korea	Breast Cancer	489	82.0 ± 36.5 m	IHC (Abcam)	IRS ≥ 2	OS, DFS	8
Sewha Kim [30]	2012	Korea	Breast Cancer	119	59.2 ± 27.9 m	IHC (Abcam)	IRS ≥ 2	OS, DFS	6
Daisuke Inoue [31]	2012	Japan	Lung Adenocarcinoma	109	1626 d (17–3366)	IHC (Santa Cruz)	≥ 10%	OS	6
Phil Rolland [32]	2007	UK	Breast Cancer	523	76 m	IHC (INC)	≥ 5%	OS	8
L-Z Xu [33]	2016	China	Breast Cancer	369	NR	IHC (NR)	NR	OS, DFS	8
Ji-Ye Kim [34]	2014	Italy	Breast Cancer	334	NR	IHC (Abcam)	≥ 30%	DFS	7
Anna M. Schläfli [35]	2016	Switzerland	Non-Small Cell Lung Cancer	446	NR	IHC (MBL)	≥ 25%	OS, DFS	7

NR: Not Reported; y: year; m: month; d: day; OS: Overall Survival; DFS: Disease-Free Survival; IRS: Immunoreactive Score.

### Correlations between p62 expression and overall survival (OS)

**Figure 2:**
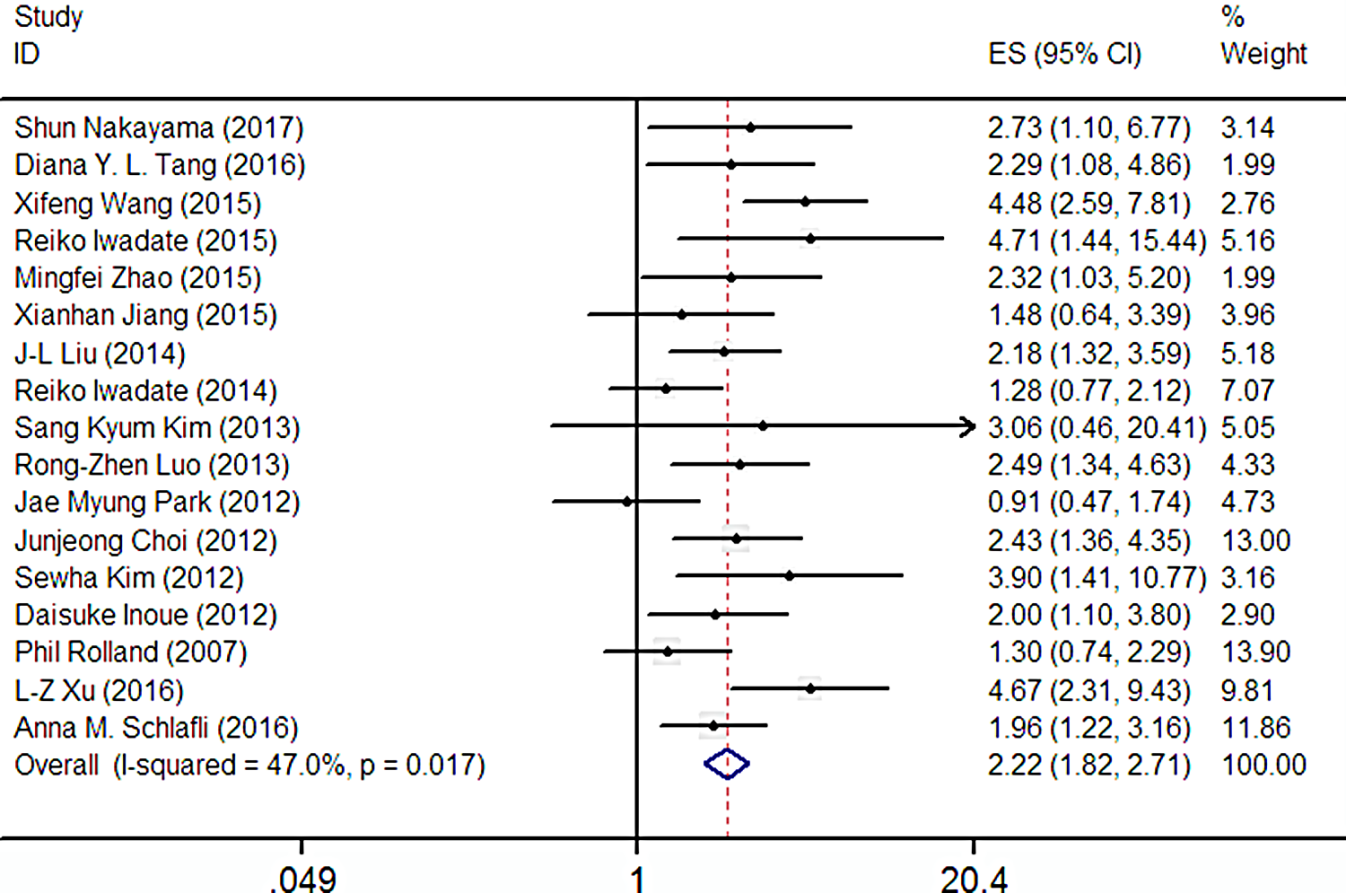
Forest plot describing the association between p62 expression and OS

17 articles included 3762 patients were selected to evaluate the relationship between p62 expression and OS. In addition, this test was analyzed using a random-effect model due to high heterogeneity (I^2^ = 47.0%). The pooled HR revealed that there was a clear correlation between the high expression of p62 and the worse OS (HR: 2.22, 95% CI: 1.82–2.71, *P* < 0.05; Figure 2) in multivariate analysis. These discoveries indicate that p62 is a prognostic factor for various types of cancer.

To minimize heterogeneity, the subgroup analyses were performed according to the ethnics (Asian or not), case number (≥ 150 or not), NOS score (≥ 7 or not), antibodies (various company), cut-off value (various scoring criteria). The pooled HRs and heterogeneities according to all these factors were presented in Table 2. Unfortunately, all these subgroup analyses demonstrated that there were no significant lower I^2^ value when the *P* < 0.05. Therefore, subgroup analysis were failed to find the origin of high heterogeneity.

**Table 2:** Associations between p62 expression and OS stratified according to the ethnics, case number, NOS score, antibodies and cut-off value

Categories	Subgroups	Ref	HR (95% CI)	Heterogeneity test (I^2^, *P*-value)
Ethnics	Asian	[16, 19–24, 26, 27, 29–31, 33]	2.69 (2.08–3.48)	37.3%, 0.085
	Not Asian	[18, 28, 32, 35]	1.48 (1.08–2.04)	39.4%, 0.176
Case Number	≥ 150	[20, 23, 24, 26–29, 32, 33, 35]	2.15 (1.69–2.72)	56.5%, 0.014
	<150	[16, 18, 19, 21, 22, 30, 31]	2.53 (1.84–3.47)	15.4%, 0.312
NOS Score	≥ 7	[16, 19, 20, 22–24, 26–29, 32, 33, 35]	2.18 (1.75–2.71)	58.1%, 0.004
	<7	[18, 21, 30, 31]	2.61 (1.70–4.00)	0.0%, 0.681
Antibody	Santa Cruz	[20–22, 24, 27, 31]	2.11 (1.50–2.96)	23.5%, 0.258
	Abcam	[19, 23, 26, 29, 30]	2.77 (1.77–4.32)	9.9%, 0.350
	MBL	[16, 28, 35]	1.72 (1.20–2.46)	59.3%, 0.086
	NR	[18, 32, 33]	2.21 (1.47–3.34)	74.2%, 0.021
Cut-off Value	IRS	[19,21–23,26, 27, 29, 30]	2.55 (1.80–3.61)	0.0%, 0.448
	Percentage	[16, 18, 20, 24, 28, 31, 32, 35]	1.73 (1.34–2.23)	34.9%, 0.150

IRS: Immunoreactive Score; NR: Not Reported.

### Correlations between p62 expression and disease-free survival (DFS)

**Figure 3:**
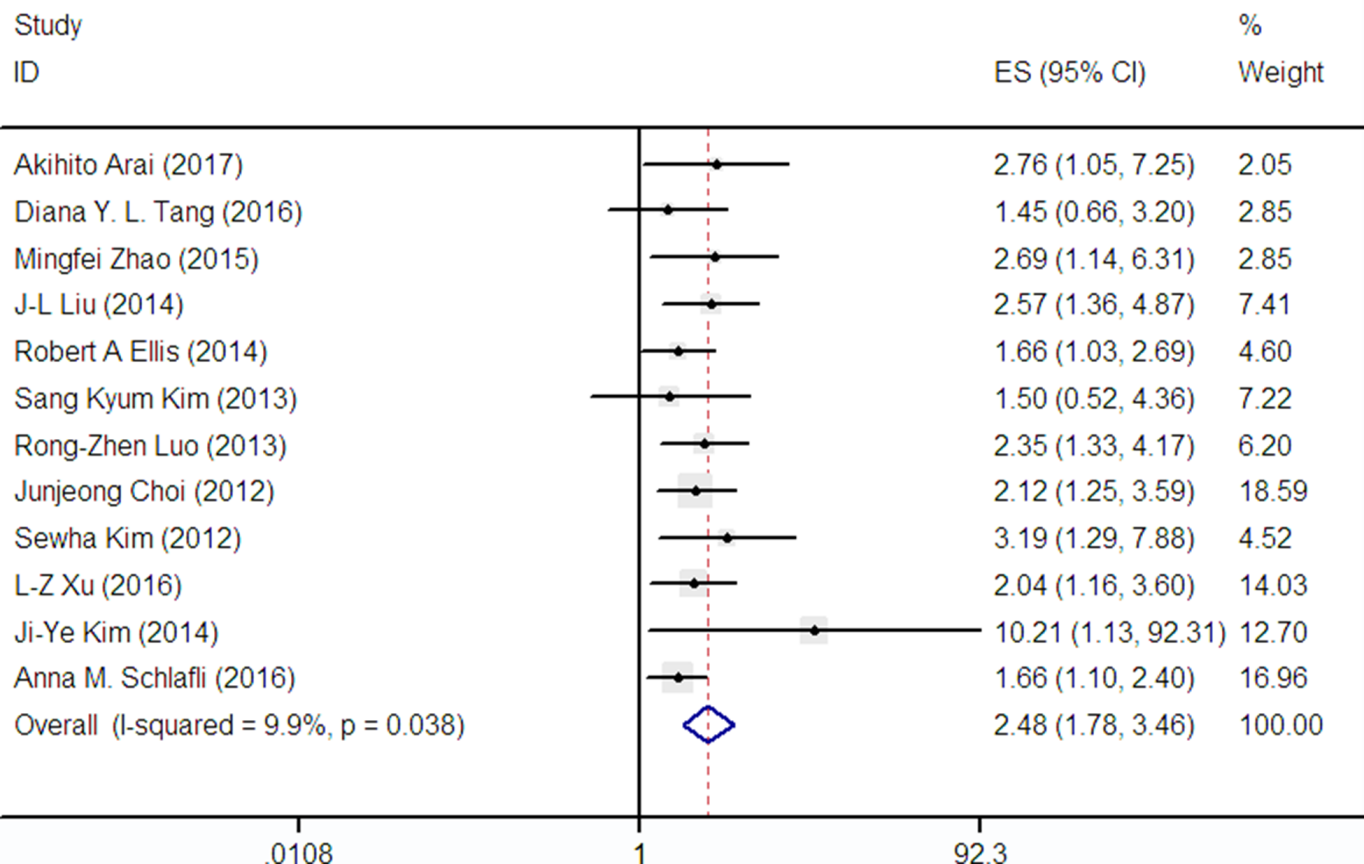
Forest plot describing the association between p62 expression and DFS

DFS was reported in 12 publications covering 2630 patients. A low heterogeneity (I^2^ = 9.9%) was observed among these studies, so we adopt a fixed-effect model to analysis. Nonetheless, the combined HR for these articles assessing p62 amplification on DFS was 2.48 (95% CI: 1.78–3.46) as shown in Figure 3, demonstrating that p62 overexpression was an indicator of poor prognosis in cancer patients.

### Correlations between p62 expression and clinicopathological parameters

As shown in Supplementary Figure 1, 14 eligible articles were used to collect the clinical and pathological parameters. Meanwhile, pooled results of the correlations were identified between the over-expressed p62 and clinicopathological features of patients with solid tumors. Our results showed that p62 overexpression was related to lymph node metastasis (RR = 1.21, 95% CI: 1.06–1.37) and clinical stages (RR = 1.27, 95% CI: 1.12–1.45), which was independent of gender (RR = 1.00, 95% CI: 0.78–1.29), tumor differentiation (RR = 0.86, 95% CI: 0.67–1.11) and tumor status (RR = 1.00, 95% CI: 0.78–1.29) (see Table 3 and Supplementary Figure 1).

**Table 3:** Meta-analysis results of the associations of p62 expression with clinicopathological parameters

Clinicopathological parameter	Ref	Overall OR (95% CI)	Heterogeneity test (I^2^, *P*-value)
Gender (male vs female)	[16, 17, 19, 21, 23, 31, 35]	1.00 (0.78–1.29)	0.0%, 0.663
Tumor Differentiation (poor VS well)	[16, 17, 19, 20, 29–32]	0.86 (0.67–1.11)	71.8%, 0.001
Tumor Size (T3-4 vs T1-2)	[17, 20, 23, 27, 29, 30, 32, 33, 35]	1.13 (0.96–1.33)	60.6%, 0.009
Lymph Node Metastasis (yes vs no)	[16, 19, 20, 27, 29–33]	1.21 (1.06–1.37)	78.6%, < 0.001
Clinical Stage (III-IV vs I-II)	[16, 17, 19–21, 23, 24, 27, 29–31, 33, 35]	127 (1.12–1.45)	84.3%, < 0.001

### Assessment of heterogeneity and sensitivity analysis

**Figure 4:**
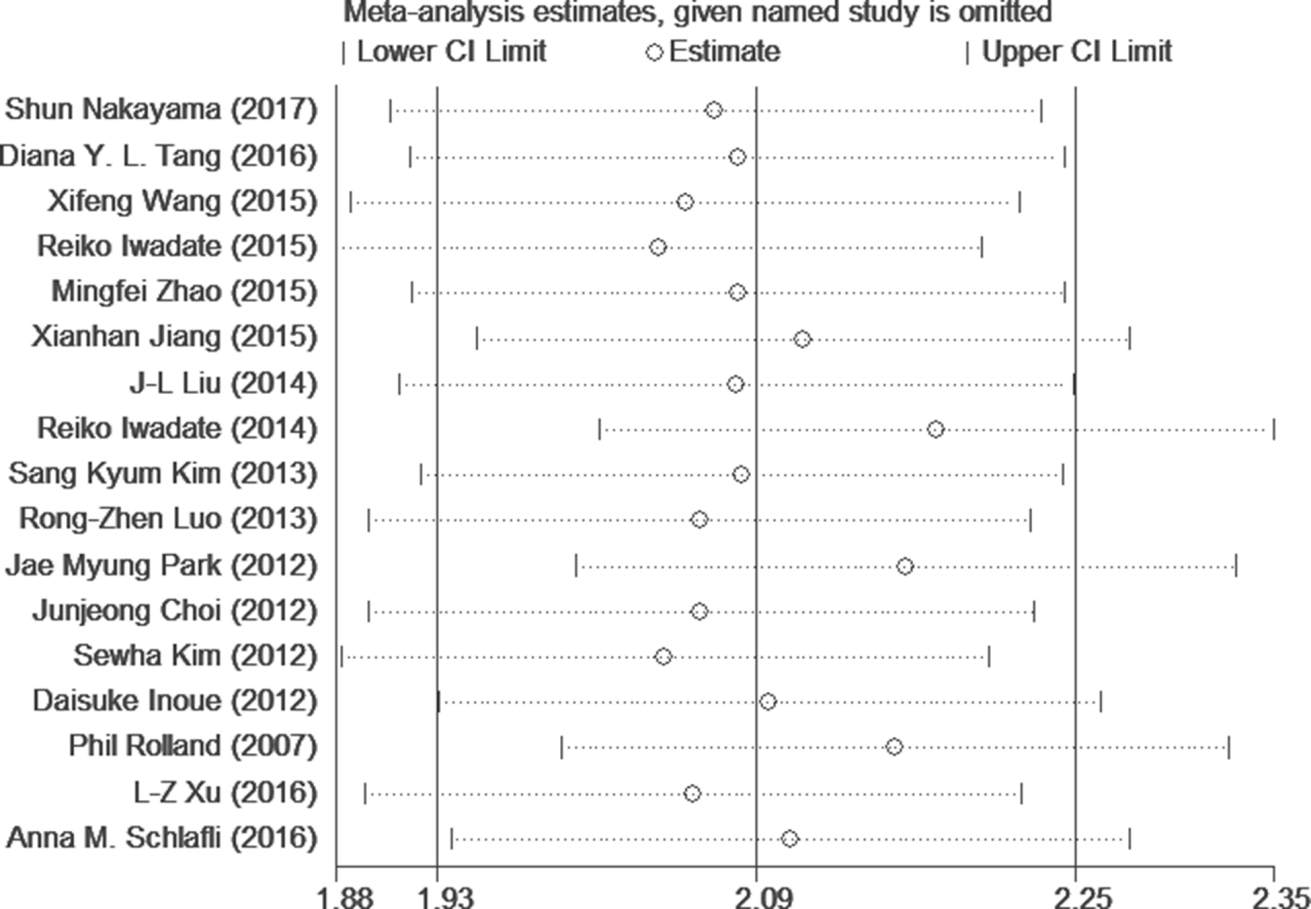
Sensitivity analysis of the OS in the meta-analysis

There was significant heterogeneity (I^2^ > 30%) among studies in OS and clinical pathological parameters analyses. Therefore, a random-effect model was adopted in these studies. A meta-regression analysis with published country, case number (≥150 or not), NOS Score (≥7 or not), antibodies (used for different companies) and cut-off value (IRS scores or Percentage) as covariates was conducted. All covariates were fit into the meta-regression model one at a time to identify potential sources of heterogeneity. However, none of these covariates were verified as a significant source of heterogeneity (Table 4). Moreover, to determine whether modifications of the included criteria affected the results, we tested this meta-analysis by a sensitivity analysis (Figure 4). The results indicated that the pooled estimates of the effect of over-expressed p62 on OS in solid tumors did not vary significantly with the exclusion of any individual studies. Also it meant that the results of this meta-analysis were stable after using the leave-one-out method.

**Table 4:** Results of meta-regression analysis exploring the source of heterogeneity with OS

Covariates	OS		
	Coef.	S.E.	*P* value
Country	-0.126	0.067	0.079
Case Number	-0.272	0.237	0.269
NOS	-0.118	0.297	0.698
Antibody	-0.004	0.110	0.970
Cut-off value	-0.510	0.192	0.081

Coef.: Coefficient; S.E.: Standard Error.

### Publication bias

**Figure 5:**
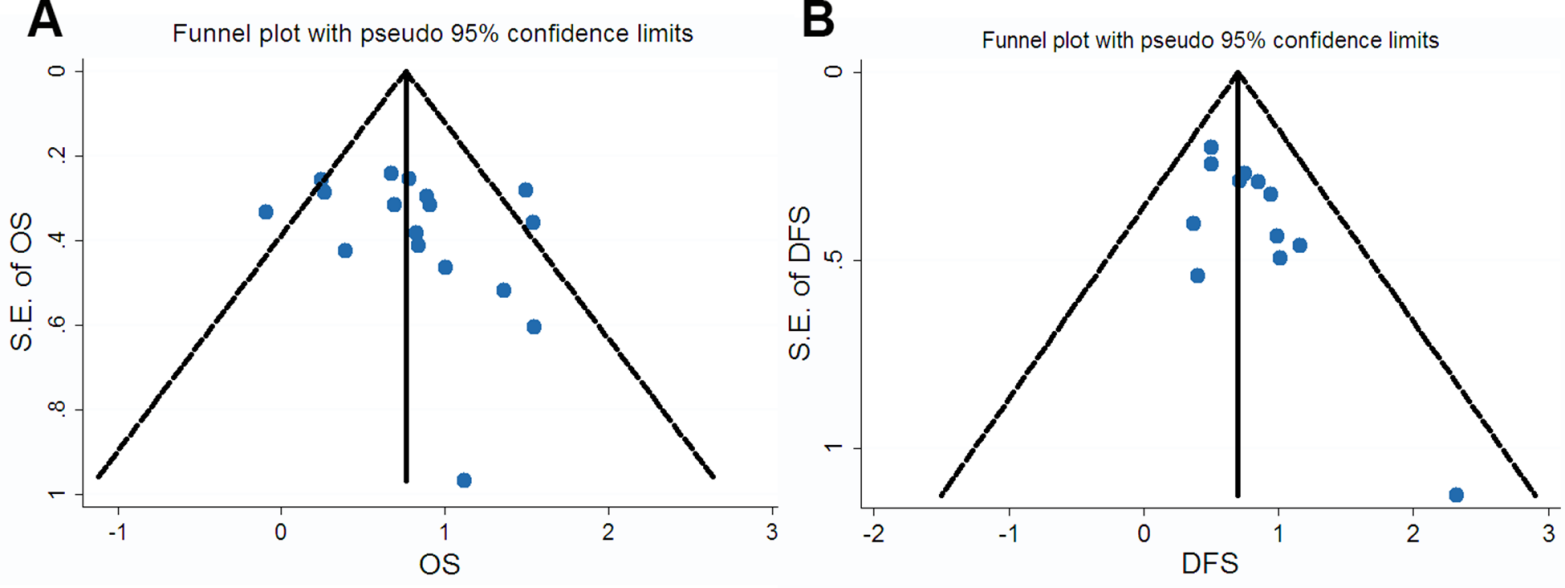
Funnel plot for the assessment of potential publication bias regarding OS (**A**) and DFS (**B**) in the meta-analysis.

We constructed Begg’s funnel plot with pseudo 95% confidence limits and Egger’s test to assess the publication bias of these applicable studies. The shapes of the funnel plots for OS, DFS and clinicopathological parameters showed no evidences of obvious asymmetry, and Egger’s test indicated the absence of publication bias (*p* > 0.05). The above results indicated that this meta-analysis was statistically reliable. Furthermore, these findings were other strong evidences to verify that p62 was a prognostic factor for cancer patients (see Figure 5 and Supplementary Figure 2).

## DISCUSSION

The meta-analysis presented herein is the first to describe all the reported studies investigating the impact of p62 expression in human tumors on prognosis. Furthermore, this analysis aimed to examine the association between p62 expression with OS and DFS of cancer patients. We combined the outcomes of 4271 cancer patients from 20 individual studies, suggesting that p62 high-expression significantly predicted poor OS (HR: 2.22, 95% CI: 1.82–2.71, *P* < 0.05) and DFS (HR: 2.48, 95% CI: 1.78–3.46, *P* < 0.05). Moreover, obvious correlations were observed between p62 overexpression and clinicopathological characteristics including lymph node metastasis (RR = 1.21, 95% CI: 1.06–1.37) and clinical stages (RR = 1.27, 95% CI: 1.12–1.45), which indicated general roles of p62 in cancer prognosis.

Our study has several strengths. The included original articles were all prospective, which greatly reduces the likelihood of selection bias and reverse causation. In addition, a large number of cases had been collected from different studies, and 4271 participants represented a large number, significantly increasing the statistical capacity of the analysis. Lastly, funnel plot and Begg’s analysis didn’t discover any publication bias, suggesting that the results has a high degree of credibility. Nevertheless, this meta-analysis also had an inherent potential limitation that should be considered. This potential limitation is the high heterogeneity between OS and different clinicopathological parameter analyses. High heterogeneity may come from the following sources: first, there were no uniform criteria for IHC evaluation and cutoff point now. The assessment of p62 expression and determination of cutoff values were based on personal judgment. Second, the results might vary with age, gender, territory, tumor grade and staging. Third, diverse methods of survival data analysis in various studies were considered as potential sources of heterogeneity.

This study analyzed the expression of p62 in various kinds of tumor tissues and evaluated its prognostic value. The results showed that the high expression of p62 was related to poor prognosis. It is noteworthy that both autophagy and inflammatory pathway for cancer development have the duality in previous research, but our analysis shows the role of p62 in the prognosis of cancer development has been clear and simple. As a scaffold protein of multiple pathways, the simple relationship between p62 and cancer prognosis can be attributed to the alternate of following pathways under high level of p62: First, overexpression of p62 can inhibit autophagy to make the accumulation and aggregation of itself, which is toxic and can promote tumorigenesis. Second, high level of p62 can activate NF-κB signaling and Nrf2 to degrade ROS respectively, which may help cancer cell evade apoptosis to promote cancer progress. Furthermore, the role of p62 in autophagy pathways is worthy of widespread attention in cancer research. Autophagy is a crucial target for cancer therapy, and most autophagy-target anticancer drugs inhibit autophagy (like rapamycin and it analogs). In contrast, as shown in laboratory research and our meta-analysis, the high level of p62-a native autophagy inhibitor, can promote carcinogenesis. It is also worthy of note that most of the current autophagy-target drugs have poor efficacy and are susceptible to drug resistance [36]. Those facts remind us that p62-autophagy-cancer relationship is not only a valuable prognosis biomarker for cancer patients, but also help scientists to reevaluate the role of autophagy in cancer, and may contribute to the development of current autophagy-target cancer therapy.

## MATERIALS AND METHODS

### Search strategy and selection criteria

We searched the relevant studies from the PubMed, EMBASE and ISI Web of Science, Nature databases using the following keywords in all possible combinations: p62, prognosis, and tumor. The last systematical search was performed on September 20, 2017. Criteria for eligibility of each study included in this meta-analysis were: (1) the correlation between p62 expression and overall survival (OS) or disease-free survival (DFS) of cancer patients; (2) the expression of p62 was detected by immunohistochemistry; (3) to provide adequate information to assess hazard ratio (HR) and 95% confidence interval (CI); (4) pathological diagnosis of various tumor types or clinicopathological features were described; and (5) to be published as a full text in the English language. Reviews, letters, comments, repetitive researches, case reports, and personal communications were excluded. Laboratory studies were also excluded if they did not provide quantitative data regarding the primary outcome measure.

### Data extraction

Two investigators (Haihua Ruan and Lingling Wang) independently reviewed each eligible publication and extracted data by a standardized data-extract form. The uniform information collected were as follows: first author’s name, publication date, the patient’s region, type of cancer, p62 detection method, number of cases, number of patients with p62-positive, follow-up times, cut-off values, and clinicopathological parameters. If the results were inconsistent, the third person would join the discussion and made the final decision [37].

### Statistical method of meta-analyses and quality assessment

All the statistical analyses were performed using Stata 12.0 (Stata Corporation, College 216 Station, TX, USA) software. Pooled estimates of hazard ratios (HR) with their 95% confidence intervals (CIs) were used to assess the relevance between p62 expression and survival outcome and clinical parameters, including gender, tumor differentiation, lymph node metastasis, distant metastasis as well as clinical stage. Multivariate HR and 95% CI were employed when both univariate and multivariate results were provided. Moreover, each of the 20 eligible studies included in our meta-analysis was assessed for quality according to the Newcastle–Ottawa Scale (NOS). The NOS score ranged from 0 to 9, and studies with NOS score ≥7 were defined as high-quality studies. The heterogeneity within studies was tested with chi squared test (Cochrane’s *Q* test) and I-squared statistical test. The random-effects model was adopted when the result of a *Q*-test (I^2^ > 30% or *P* < 0.05, high heterogeneity). Furthermore, evidence of publication bias was used by Begg’s funnel plot and Egger’s test [38, 39]. *P* < 0.05 was considered to be statistically significant in this analysis.

## SUPPLEMENTARY MATERIALS FIGURES


