# Supplementary material for: The prognostic value of p62 in solid tumor patients: a meta-analysis
Source: Oncotarget. 2017 Dec 7;9(3):4258–66. doi: 10.18632/oncotarget.23101 (PMC5790537; doi:10.18632/oncotarget.23101)
Supplement: Supplementary file 1 [file oncotarget-09-4258-s001.pdf]

The prognostic value of p62 in solid tumor patients: a meta-analysis

SUPPLEMENTARY MATERIALS

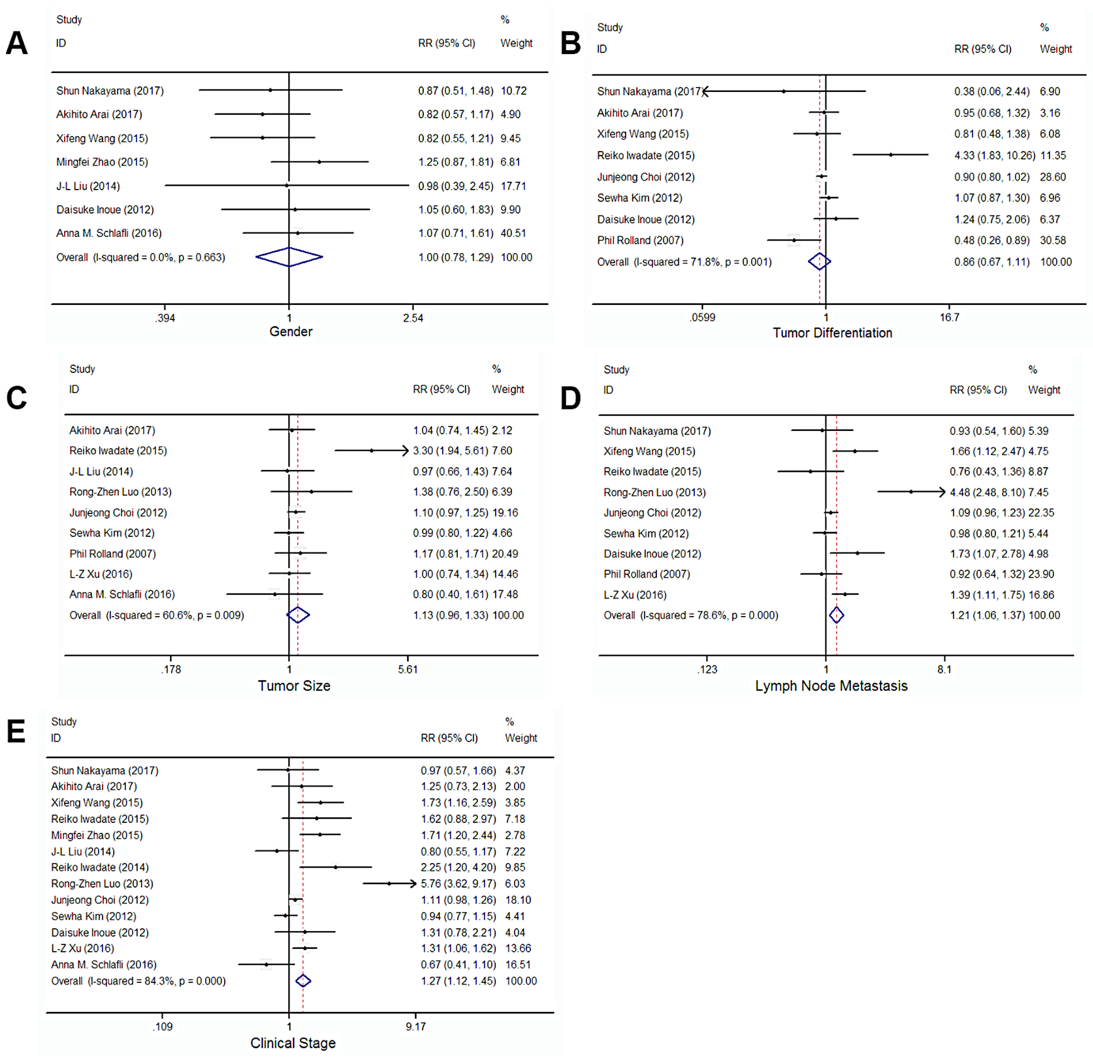

Supplementary Figure 1: Forest plot describing the association between over-expressed p62 and clinicopathological parameters, including Gender (**A**), Tumor Differentiation (**B**), Tumor Size (**C**), Lymph Node Metastasis (**D**), and Clinical Stages (**E**).

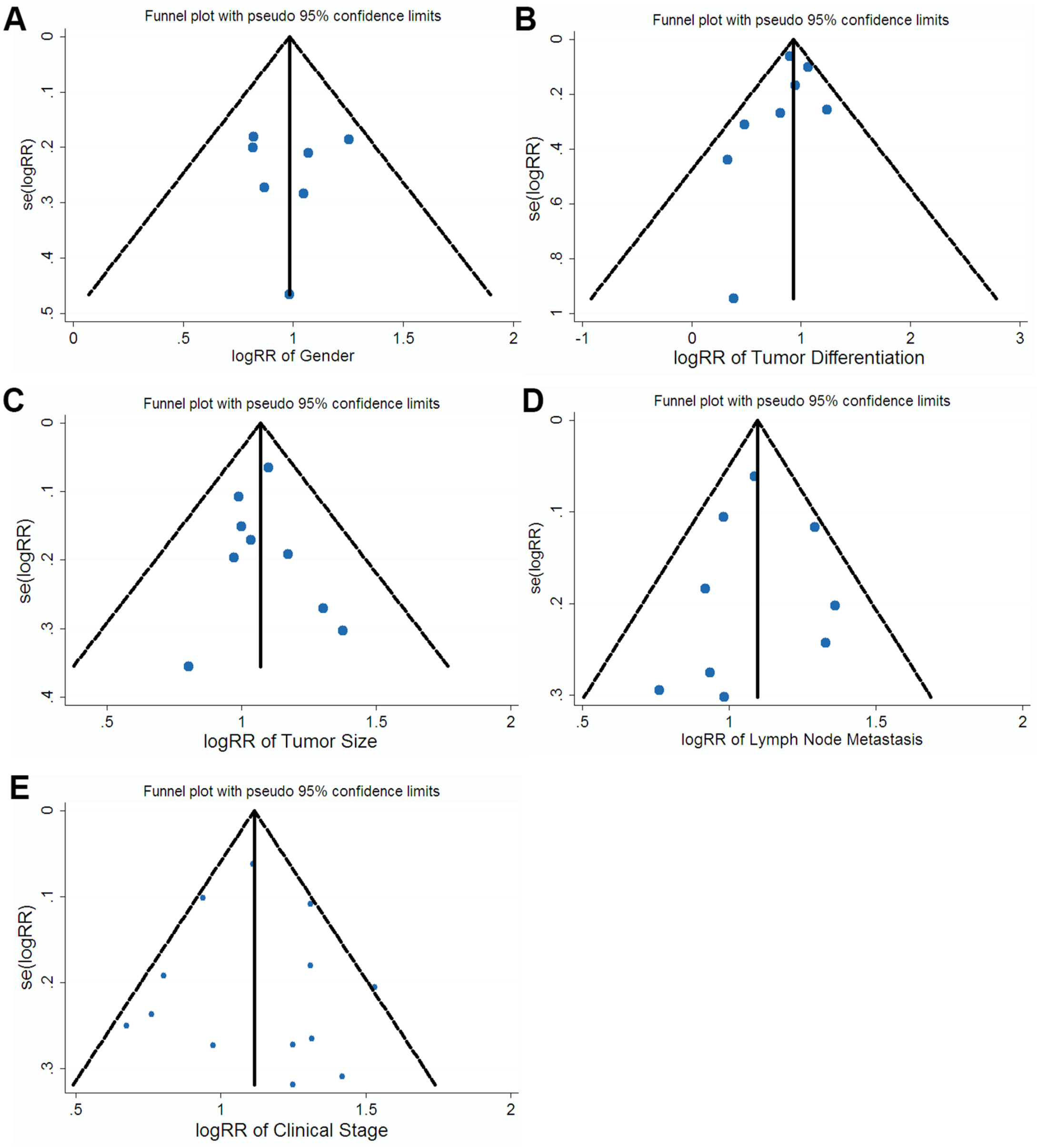

Supplementary Figure 2: Funnel plot for the assessment of potential publication bias regarding clinicopathological parameters in the meta-analysis. Clinicopathological parameters including Gender (**A**), Tumor Differentiation (**B**), Tumor Size (**C**), Lymph Node Metastasis (**D**), and Clinical Stages (**E**).
